# An incidental finding of additional vascular compression of the brachial plexus during ultrasound-guided anterior scalene bupivacaine injection for neurogenic thoracic outlet syndrome: addition of hydrodissection—a case report

**DOI:** 10.1186/s13256-026-06237-y

**Published:** 2026-07-01

**Authors:** Omar Itani, Youssef Jamaleddine, Majed Ali, Emanuel-Youssef Dib, Mohammad Badra, Noura Hanna, Ramzi Moucharafieh, Mohammad Nedal Jomaa

**Affiliations:** 1 Department of Anesthesia, Clemenceau Medical Center, Beirut, Lebanon; 2 Department of Anesthesia, Faculty of Medicine, University of Balamand, Balamand, Lebanon; 3 Department of Orthopedics, Faculty of Medicine, Lebanese American University, Beirut, Lebanon; 4 Department of Orthopedics, Clemenceau Medical Center, Beirut, Lebanon; 5 Department of Orthopedics, Faculty of Medicine, University of Balamand, Balamand, Lebanon

**Keywords:** Dorsal scapular artery, Thoracic outlet syndrome, Anterior scalene injection, Hydrodissection, Bupivacaine, Brachial plexus

## Abstract

**Background:**

Bony, muscular, and fibrous abnormalities in the thoracic outlet are implicated in the pathogenesis of neurogenic thoracic outlet syndrome (nTOS). Compression of the brachial plexus by the dorsal scapular artery (DSA) is rarely reported and is often diagnosed incidentally during cadaveric dissection or surgical decompression.

**Case presentation:**

We present the case of a 51-year-old Middle Eastern female who presented with chronic left cervicobrachialgia associated with left-hand paresthesia and weakness. Physical examination supported the diagnosis of nTOS. Imaging revealed an elongated C7 transverse process and aberrant and accessory muscular slips of the anterior scalene muscle. During ultrasound-guided anterior scalene bupivacaine injection, a large blood vessel was observed to dynamically impinge over the C7 nerve root, and hydrodissection using 10 ml of normal saline was added to the intervention. On subsequent computed tomography angiogram and retrospective review of magnetic resonance imaging, this artery was identified as the DSA unusually arising from the first part of the subclavian artery. The patient experienced a dramatic 80% symptom relief 1 week after the intervention. A further 10% relief was experienced following subsequent pectoralis minor bupivacaine injection. nTOS-directed physiotherapy was initiated. Persistence of improvement was observed 3 months after the injections. Recurrence of symptoms occurred starting from the eighth month post-intervention.

**Conclusions:**

This case highlights the possible contribution of the DSA to nTOS, the diagnostic value of a dynamic imaging modality, and the incorporation of hydrodissection in the decision-making process. Diagnostic muscle injections using Bupivacaine and neurovascular hydrodissection using normal saline lead in this case to 8 months of symptom relief and allowed us to locate the culprit structures.

**Supplementary Information:**

The online version contains supplementary material available at https://doi.org/10.1186/s13256-026-06237-y.

## Background

Neurogenic thoracic outlet syndrome (nTOS) is caused by extrinsic compression of a part of the brachial plexus (BP) as it passes through a long musculo-osseous corridor comprising the interscalene triangle, the costoclavicular space and the sub-coracoid retro-pectoralis minor space [1]. The pathomechanism involves acquired or traumatic factors associated with one or more predisposing congenital anomalous structure(s) in the outlet, such as a rudimentary cervical rib, an innate fibrotic band, an accessory muscle slip, and/or an anomalous muscle insertion [1–3]. Mixed culprit anomalies at more than one location can coexist in the same patient, complicating the diagnostic and therapeutic processes [1, 3, 4].

Vascular structures traversing through the BP, without necessarily having an impinging course on neural structures, are commonly observed by anesthesiologists during ultrasound-guided regional blocks [5]. The identification of a vascular structure as a compressing or impinging culprit is a relatively recent discovery in the thoracic outlet literature [6]. Almost all relevant reports identify this structure as the dorsal scapular artery (DSA), which causes nTOS by riding over and compressing a nerve root or a BP trunk. These reports are mostly incidental findings during cadaveric dissections [6, 7] or during routine surgical decompression procedures of the thoracic outlet [7–9].

In this report, we present a case of nTOS in a female who had a combination of anomalies in the thoracic outlet. During ultrasound-guided anterior scalene test injection, the impinging course of the DSA was identified and addressed with hydrodissection. This case highlights the diagnostic importance of a dynamic imaging modality in revealing neurovascular conflict in real time, the potential contribution of DSA to nTOS, and the diagnostic workup and conservative management of multifactorial nTOS using targeted injections.

## Case presentation

A 51-year-old left-hand-dominant Middle Eastern female presented to our clinic for a 2-year history of atraumatic left cervicobrachial, pectoral, axillary and scapular pain associated with positional, nonradicular paresthesia in the medial forearm, the palm and the volar aspects of the medial four fingers. In addition, she described ipsilateral hand weakness and “clumsiness”. There was no resting or positional arm or hand swelling, cyanosis, erythema, or pallor. She also complained about intermittent bouts of dizziness, blurred vision, teeth clenching, and occipital and bitemporal headaches. Prior to the onset of her symptoms, she had had a negative past medical history. There was a negative family history for a similar presentation. The patient came from a highly educated background with a high socioeconomic status, and she denied any history of depression, anxiety, or any other psychiatric illness.

Long-term analgesics, NSAIDs, muscle relaxants, magnesium supplements, physiotherapy and chiropractor sessions failed to provide sustainable or predictable relief.

Physical examination revealed left scapular malposture with mild protraction, anterior tilt and internal rotation. The inferior scapular angle was prominent without winging during motion. There were full unrestricted active and passive ranges of motion of the left shoulder and neck, but motion was painful due to muscle spasms. Left shoulder exams for subacromial impingement, rotator cuff pathology, glenohumeral arthritis/instability, acromioclavicular arthritis/instability and biceps pathology were all negative.

The result of the *Adson* test [10] was negative. The radial pulse did not diminish during 180° of lateral elevation. There was no change in hand color or temperature during overhead maneuvers or prolonged limb elevation. No collateral venous circulation could be observed in the shoulder or pectoral region following repeated shoulder circumduction exercises.

Exerting supraclavicular interscalene pressure yielded localized tenderness and discomfort and triggered instant paresthesia in the volar aspects of the medial four fingers. Taking this pressure off stopped the paresthesia (positive *Tinel* sign [4, 10]).

Palpating and percussing the pectoralis minor insertion on the medial coracoid yielded this distal paresthesia as well but to a lesser intensity. The coracoid tip is tender to palpation.

Finger and medial forearm paresthesia appeared during shoulder abduction at 90° and remained through 180° (positive *Wright* test [10]). *The elevated arm stress test* (*of Roos*) [4, 10] was positive; the patient collapsed her arm at 60 s and could not last longer. *The modified upper limb tension test* (of *Elvey*) [4, 10] was positive. The spurling test triggered neck pain but did not reproduce distal tingling. Provocative exams for carpal tunnel syndrome, cubital tunnel syndrome and lacertus syndrome were negative. The patient had 4 + /5 strengths in the left interossei and in the left thenar and hypothenar groups, with no evidence of atrophy (with 4 + grade defined as: patient resists well but yields under maximal effort, indicating *subtle* weakness). There were normal biceps, triceps and brachioradialis reflexes on the left side. There were no signs of upper motor neuron lesions. There was no ataxic gait.

A review of past charts revealed a normal dynamic duplex ultrasound of the vertebral, carotid, axillary, brachial, radial and ulnar arteries dated to a few months prior to presentation. The axillary artery and its branches had triphasic waveforms and normal velocities with no evidence of significantly decreased flow distally in the 180° arm position.

Magnetic resonance imaging (MRI) of the brain conducted 2 years prior to presentation was normal.

A review of recent blood workup revealed normal HBA1c (4.7%), TSH (0.81 mIU/ml), free T3 (2.54 pg/ml) and free T4 (1.04 ng/dl) levels. The ESR and CRP levels were within normal ranges.

**Fig. 1:**
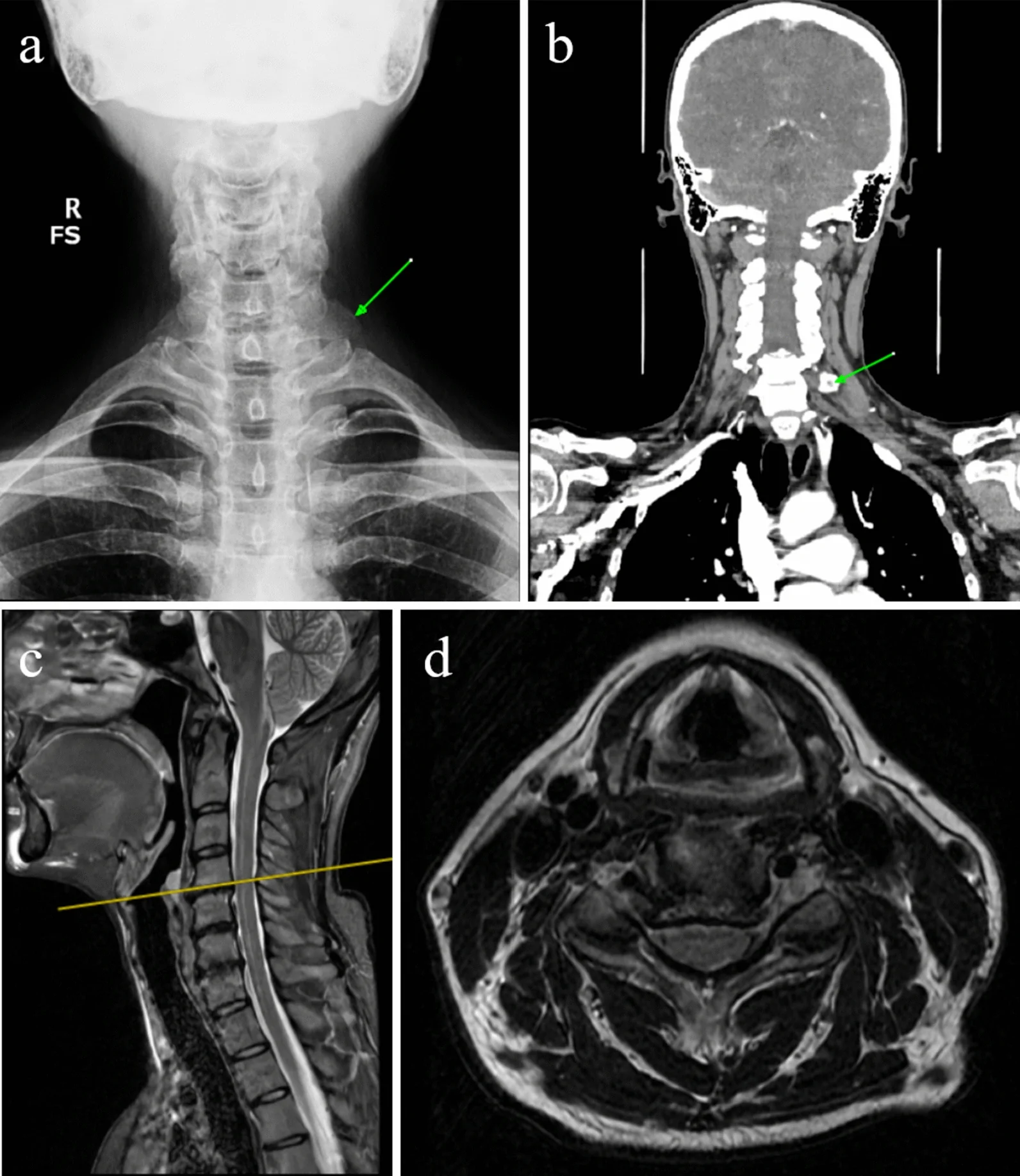
Anteroposterior cervical X-ray acquired following the initial presentation (**a**) and coronal reconstruction of a cervical computed tomography arteriogram acquired following the therapeutic injections (**b**) showing elongated and hypertrophic left transverse process of C7 (arrow). The process is seen violating the left interscalene space and contacting neuromuscular structures. **c**, **d** Sagittal T2 FSE STIR (**c**) and corresponding Axial T2 FSE (**d**) sections showing severe C4–C5 foraminal narrowing on the right side and a mild narrowing on the left side. The patient’s symptomatology is on the left side

**Fig. 2:**
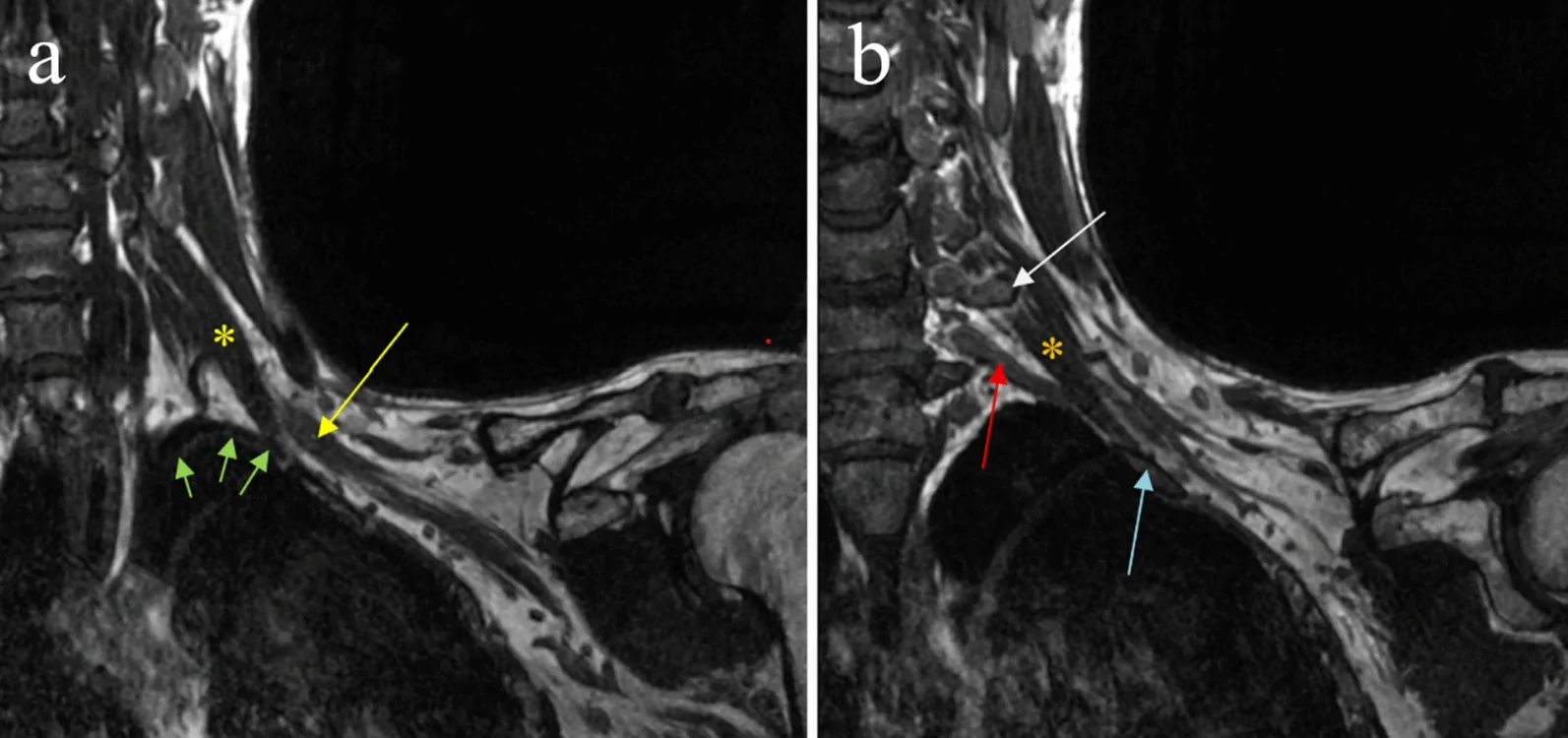
Coronal FIESTA sequence. Section **a** shows a second anomalous insertion (yellow asterisk) of anterior scalene muscle on the first rib posterior to the subclavian artery (the border of the artery is evident in this section and is marked with green arrows). This abnormal muscular band crosses anterior to the brachial plexus (yellow arrow), narrowing the interscalene space. Normally, there are no muscular insertions between the subclavian artery and the trunks of the brachial plexus. Section **b** accessory scalene muscle, called scalene minimus (orange asterisk), originating from the hypertrophic C7 transverse process (white arrow) and inserting on the first rib (blue arrow). C8 nerve root (red arrow) is seen just traversing under scalene minimus

Imaging revealed an *elongated, hypertrophic C7 transverse process* on the left side (Figs. 1, 2, 5). A cervical MRI revealed cervical spondylosis. There was mild degenerative narrowing of the left C4‒C5 intervertebral foramen without frank compression on the exiting nerve root (Fig. 1). The other left-sided foramina were patent.

MRI of the BP ruled out brachial neuritis. *A second anomalous insertion of the anterior scalene muscle* was observed on the first rib *posterior* to the subclavian artery. BP MRI also revealed a left *accessory scalene minimus* muscle originating from the hypertrophic transverse process of C7 and inserting on the first rib (Fig. 2) adjacent to the anomalous insertion of the anterior scalene muscle. These abnormal muscle bands resulted in narrowing of the left interscalene triangle.

Nerve conduction studies of the upper extremities were done along the median, ulnar and medial brachial cutaneous nerves bilaterally. All the parameters were normal and symmetrical (motor distal latency, compound muscle action potentials, sensory nerve action potentials, and sensory and motor nerve conduction velocities) except for a decrease in sensory conduction velocity of the left median nerve, with moderate delay in the MDL of both median nerves, more on the left side. The electrophysiologist considered this to be mild bilateral carpal tunnel syndrome, more pronounced on the left side.

**Fig. 3:**
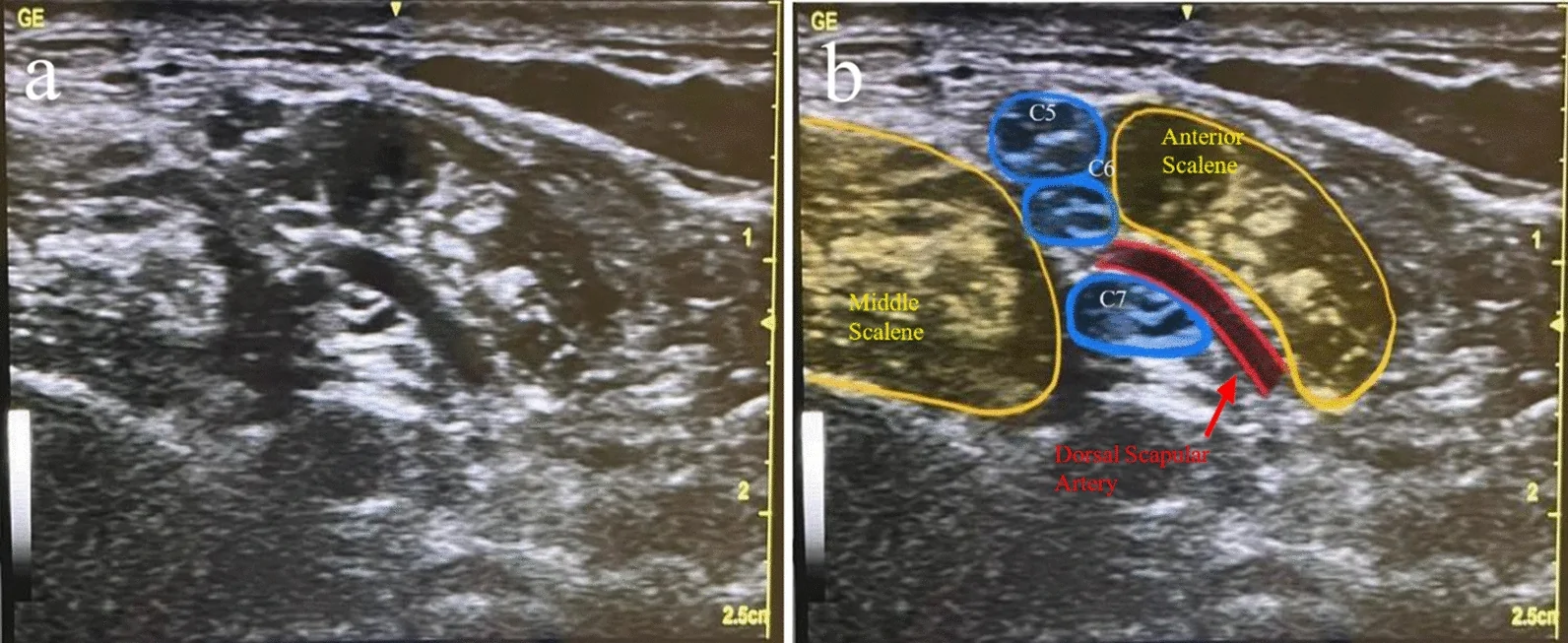
Unannotated (**a**) and annotated (**b**) ultrasound representation of DSA traversing between C6 and C7 nerve roots and impinging on the C7 nerve root

**Fig. 4:**
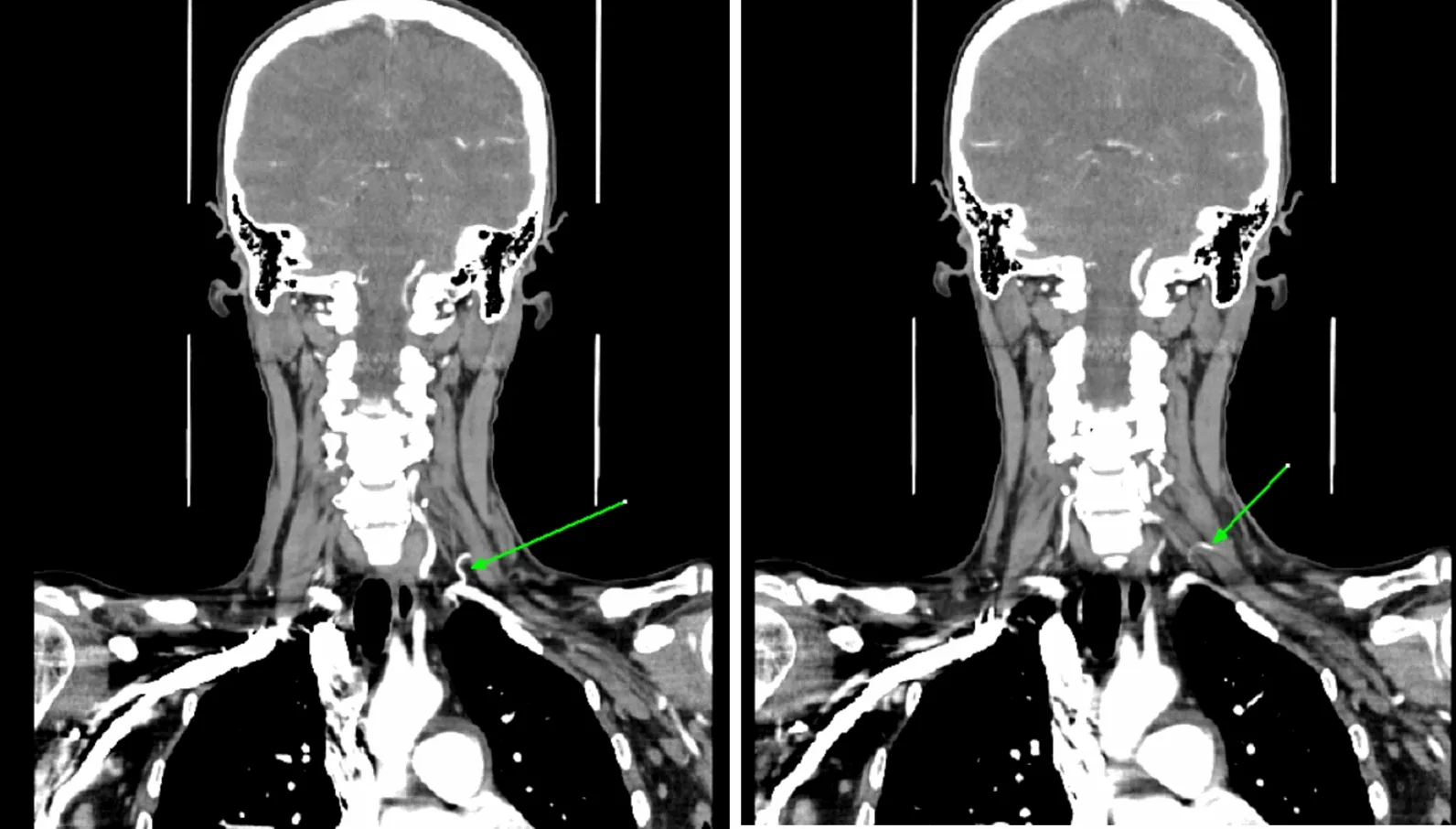
Coronal reconstruction of computed tomography angiogram acquired after the injections showing a large prominent left DSA, originating directly from the first part of the subclavian artery and taking a tortuous course into the interscalene space

**Fig. 5:**
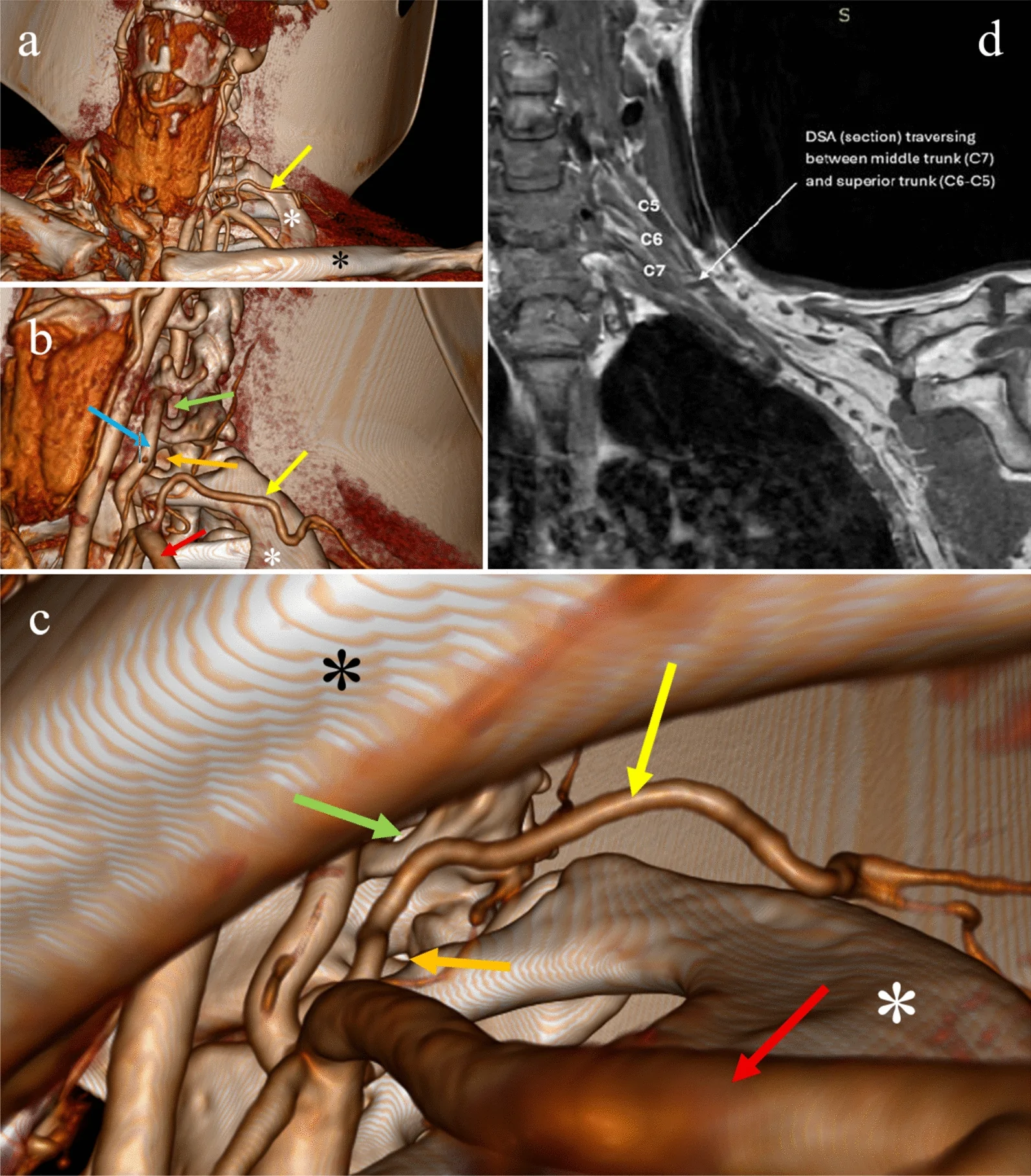
3D reconstruction of computed tomography angiogram **a**–**c** acquired after the injections showing the large, tortuous prominent DSA. **a**, **b** supraclavicular views. **c** infraclavicular view through the costoclavicular space. The MRI was analyzed retrograde after the injections, and the DSA relationship to the plexus could be appreciated (**d**). Yellow arrow: DSA. Red arrow: subclavian artery. Green arrow: C6–C7 foramen. Orange arrow: C7–T1 foramen. Blue arrow: vertebral artery. White asterisk: first rib. Black asterisk: clavicle

Based on the physical examination—especially the positive Tinel sign over the interscalene space—the absence of a radiculopathy or distal median mononeuropathy pattern, and the thoracic outlet anatomic osseous and fibromuscular anomalies discovered on imaging, a probable diagnosis of nTOS was considered (Figs. 3, 4 and 5).

**Fig. 6:**
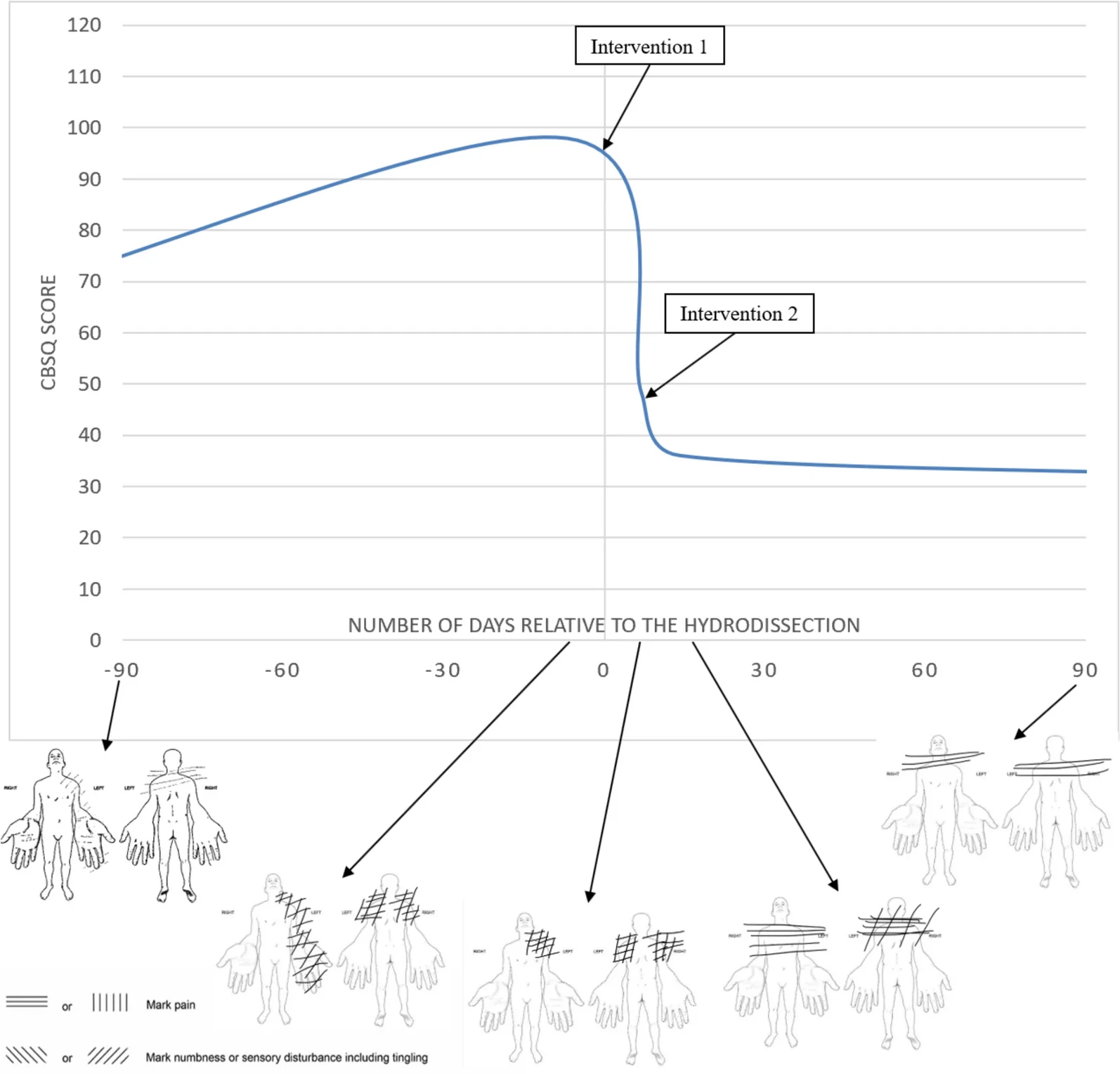
Change of the cervicobrachial symptom questionnaire (CBSQ) score from presentation (3 months prior to the first intervention) to 3 months after the first intervention. The first intervention comprised of anterior scalene muscle Bupivacaine injection in addition to hydrodissection of the DSA. The second intervention was pectoralis minor muscle Bupivacaine injection. nTOS-directed physiotherapy followed the interventions. The progression of sensory symptoms is shown at the bottom of the graph as part of the CBSQ. These maps were hand-completed by the patient at each timepoint. The cervicobrachial symptom questionnaire (CBSQ) [4, 10] is a patient-reported Outcome Measure in the form of a 14-item survery scored on a 0–120 scale (the higher the score, the worse the symptoms). It was developed for evaluation of patients with nTOS and related disorders to measure functional disturbances resulting from performance of certain activities. We calculated the CBSQ score at presentation (score = 75/120), 1 week prior to the first intervention (98/120), 1 week after the first intervention (48/120), 1 week after the second intervention (36/120), and 3 months after the interventions (32/120)

The patient delayed her medical treatment for 3 months because of travel commitments. During this time, her symptoms worsened (Fig. 6). Then, she presented to continue nTOS workup.

Our protocol for approaching nTOS includes ultrasound-guided *diagnostic* test injections of the anterior scalene and pectoralis minor muscles using a local anesthetic [10], which are spaced 1 week apart, and recording the patient’s response to these injections. We chose to use bupivacaine, rather than the usually used lidocaine, as the injected molecule to offer the possibility of a long-term therapeutic effect of the injections. We defined a positive test injection as > 50% immediate amelioration of symptoms. We also value injections as surgical prognostic predictors, with positive test results predicting a good prognosis following surgical decompression of the thoracic outlet [10].

During the first intervention (*intervention 1*), the patient was scheduled to receive anterior scalene muscle injection under ultrasound. During this procedure, a large prominent vessel was found to traverse between the C6 and C7 nerve roots. On dynamic testing, the vessel was observed to impinge on the neural structures. 3 ml of Bupivacaine 0.25% was injected into the belly of anterior scalene, distributed into two sites (middle part of the muscle adjacent to C5–C6 nerve root level, and lower part of the muscle adjacent to C7–C8 nerve root level). In addition to anterior scalene muscle injection, hydrodissection was performed using 10 mL of normal saline to separate the vessel from the nerve roots. The patient did not have any immediate or late adverse effects.

To confirm the suspected vascular anomaly, a CT angiogram was performed the second day after intervention 1. A large prominent left DSA was identified, originating directly from the first part (an unusual origin) of the subclavian artery and taking a tortuous course between anterior and middle scalene muscles, traversing the plexus trunks (Figs. 4 and 5). A retrospective analysis of preintervention BP MRI revealed the same relationship.

**Fig. 7:**
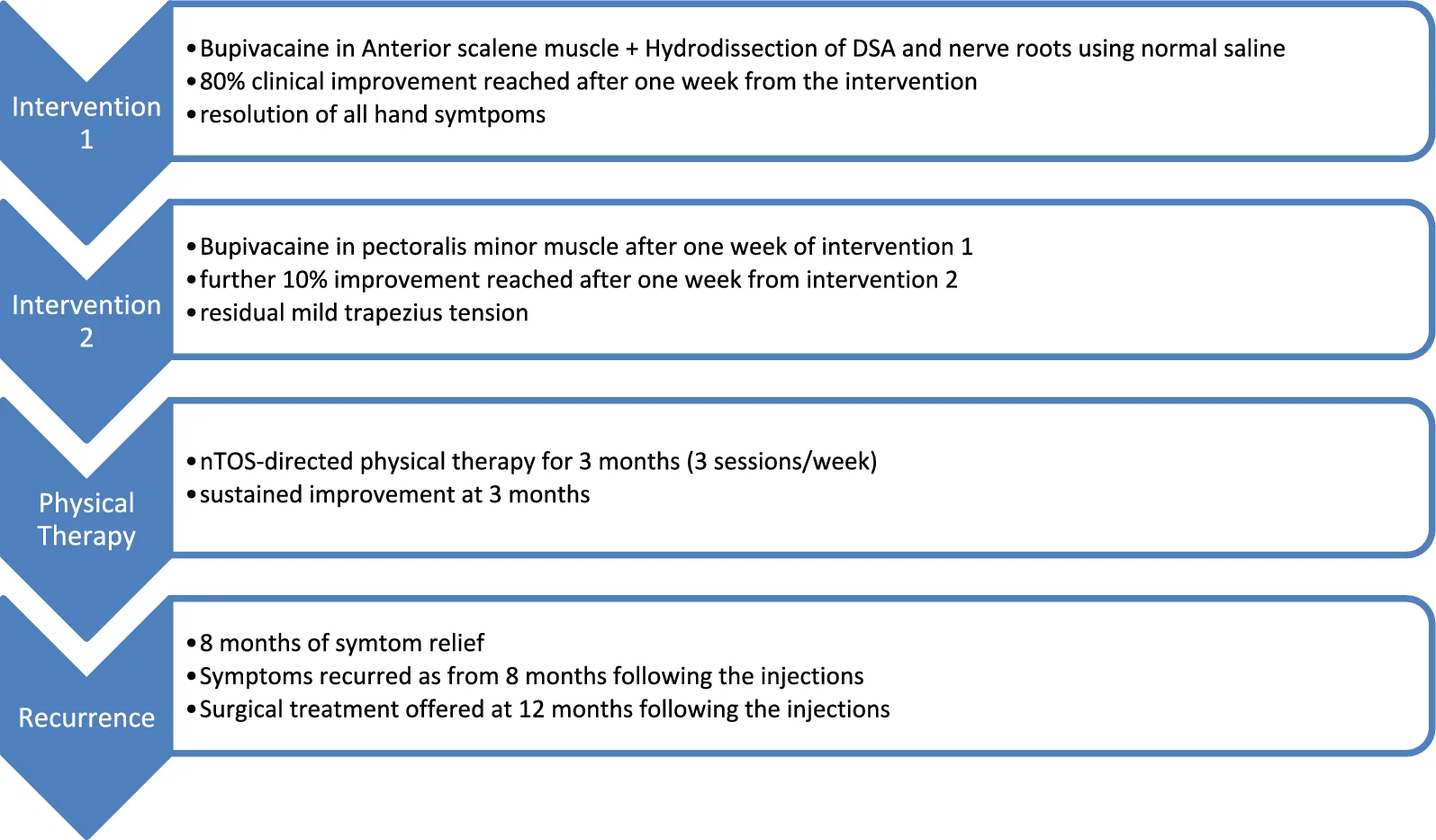
Summarized timeline showing key steps of management and follow-up

The patient-reported 80% subjective symptom amelioration 1 week following this first intervention. Hand and forearm symptoms improved completely, while there was some residual pain in the shoulder and neck area. She then underwent a second intervention (*intervention 2*) with a pectoralis minor muscle injection, 1 week after intervention 1. 3 ml of Bupivacaine 0.25% was injected into the pectoralis minor belly, distributed into three sites (lateral, middle, and medial thirds of the muscle). A further 10% subjective amelioration was achieved with no adverse effects reported. Residual symptoms were mild stiffness and intermittent pain in the trapezial region. She then received an nTOS-directed physiotherapy program for 3 months. We followed her 3 months after the injections, and she was satisfied with the complete resolution of hand symptoms and the significant reduction in pain in the neck, chest and arm areas. She had mild persistent trapezial tension, which she managed with monthly massage sessions. Strength in her left hand has improved to 5/5 (with grade 5 defined as: patient resists fully without yielding, indicating no weakness). A graphical representation of the patient’s journey is presented in Fig. 6. The patient was instructed to improve work and leisure ergonomics to maintain the corrected scapulothoracic posture. She was counselled to undergo surgical decompression if symptoms recurred or worsened [4, 10]. A summarized timeline for follow-up is shown in Fig. 7. Eight months following the injections, the patient called our clinic to report the recurrence of the same symptoms she had before the injections. She said she enjoyed months of pain relief, but her condition returned to the initial state. We advised her to undergo a new course of physical therapy coupled with an anti-inflammatory and a muscle relaxant. She did not improve on this new course. After 12 months following the injections, Tinel sign was reproduced when palpating the interscalene area. We advised the patient to undergo surgical decompression. She refused and demanded the same injection treatment. We did not offer her the injections again and explained to her that the injections are diagnostic, and that there is no evidence in medical literature on their long-term therapeutic effects. We are using bupivacaine instead of lidocaine to provide a long-term therapeutic potential in addition to the diagnostic role of the anesthetic, but the scientific evidence behind this practice is not available at this time.

## Discussion

This case involves nTOS precipitated by a combination of osseous and muscular aberrancies in the interscalene space. What was peculiar about this case was the participation of the DSA in the neural compression process. DSA emerged from the *first part* of the subclavian artery. Among the three parts of the subclavian artery, the first part is the rarest origin for DSA [11]. To the best of our knowledge, this is the first report that shows the impinging course of DSA, a usually innocuous artery, on imaging prior to surgical exploration. Other reports are incidental findings during cadaveric dissections [6, 7] or during routine surgical decompression [7–9] of the thoracic outlet.

An elongated C7 transverse process is present in approximately 4.8% of the general population; this percentage increases to approximately 50% in TOS patients [3]. In females with an elongated C7 transverse process, which lies *posterior* to the plexus, the concomitant presence of fibromuscular aberrancies which lie *in front*/*through* the plexus can be approximately 70%, creating a “scissors-like” impingement phenomenon on the BP [3]. In other words, the BP is sandwiched and compressed between two coexisting congenital anomalies. We postulate that the entrapment of the lower plexus in our patient was due to this phenomenon, accentuated further by the tethering effect of the DSA, which originates from a more proximal part of the subclavian artery than usual and rides over the C7 nerve root. This tethering effect limits the mobility of the plexus below C7, restraining it between the “blades of the scissors”. On the basis of a microanatomical fascicular topography study [12], the part of the C7 nerve root contacted by the pulsating DSA in our patient, best appreciated by ultrasound (Fig. 3), corresponds to a fascicle that will later run in the *lateral root of the median nerve*. This may explain why the patient presented with median nerve sensory symptoms in the palm and hand. This topographic finding also explains why she does not have the classical C7 radiculopathy observed in disc hernias or foraminal stenosis patients.

Multiple months of appropriate conservative management is the first-line treatment for nTOS [4, 10]. Our patient achieved rapid relief with ultrasound-guided anterior scalene muscle block and hydrodissection of the DSA from the roots of the plexus. She experienced further modest improvement with a pectoralis minor block. This finding indicates that the main problem is supraclavicular and that adaptive scapular malposture contributed only slightly to her pathology, probably secondarily. Anterior scalene bupivacaine injection relaxed the muscle, including its aberrant insertion, relatively causing the “opening of the scissors”. Hydrodissection is a technique that effectively releases entrapped nerves from surrounding muscles, fascia or adjacent structures, alleviating compressive neuropathy [13]. In our case, 10 mL of saline was sufficient to mobilize the DSA away from the C6 and C7 nerve roots, thus having a major and dramatic root decompressive effect and a complementary inferior plexus mobilization effect. Hydrodissection is emerging as a valuable option in TOS and other entrapment neuropathies, offering a minimally invasive solution that can be repeated if necessary. Recent reports have documented its use not only in neurogenic but also in vascular TOS to free the subclavian vessels and the involved plexus [13, 14].

Nambyiah et al. [5] investigated the number and distribution of arteries within the BP territory on both sides of 200 volunteers using ultrasound. At least one artery associated with the BP was identified in more than 90% of the subjects. Seventy-two percent of the arteries branched off the subclavian artery, and 45% passed in the upper zone of the plexus, bordering inferiorly to the middle trunk, as in our case.

DSA is a separate vessel that originates directly from the second or third part of the subclavian artery nearly 70% of the time and from the thyrocervical trunk nearly 30% of the time [5, 7, 11]. When it has a subclavian origin, it predominantly pierces *through* the plexus, more commonly between the upper and middle trunks (40–46%), as in our case, than between the middle and inferior trunks (23–44%) [7, 11]. When it branches from the thyrocervical trunk, it tends to pass *above* the plexus [7]. A DSA originating from the *first part* of the subclavian artery, as in this case, was reported in only one of the 178 thoracic outlets in a cadaveric study [11].

Syndromic conflicts between a vessel and a nerve are well-documented throughout the body, such as in trigeminal neuralgia or in some cases of Guyon canal syndrome or radial tunnel syndrome (leash of Henry). There are rare sporadic cases in which subclavian branches other than the DSA result in compression of neural structures, such as a vertebral artery looping around a nerve root [15] or a transverse cervical artery causing phrenic nerve palsy [16]. A similar neurovascular conflict at the level of the lumbosacral plexus caused by the superior gluteal vessels has also been documented [17].

Neurovascular entrapment sites are uncovered throughout the body via meticulous surgical exploration. Preoperative diagnosis remains challenging. Few clinical reports have shown intraoperative surgical evidence of clear and severe impingement between the DSA and the inferior trunk [7, 8], middle trunk [8], or upper trunk [9] in patients with nTOS. These patients were treated with ligation and cutting of the vessel, and immediate postoperative relief of symptoms was observed.

Multifactorial pathogenesis of nTOS may be the rule rather than the exception [1, 3, 6]. Instead of looking for one causative anatomic factor, probably the syndrome results as an interplay between several anatomic and dynamic factors. This report is limited by the fact that there were three anatomic lesions which were visualized on multiple static and dynamic imaging modalities instead of having them visualized on a live surgical field. An appreciation of the *amount* of compression that each culprit exerts is, therefore, not feasible preoperatively. However, the postulated model of interplay between these culprits is plausible and explanatory, and it fulfills our aim of having as much of the complex pathology *diagnosed before surgical exploration*. This model helped us make therapeutic decisions that maximized nonsurgical treatment in a single case who achieved a complete resolution of hand symptoms and an almost complete resolution of neck and shoulder symptoms for 8 months. It is beyond the scope of this paper to study the therapeutic effects of individual injections, and we admit the confounding between the anterior scalene muscle injection and the neurovascular hydrodissection during *intervention 1*. This report is rather focused on the diagnostic and decision-making processes of a complex, multifactorial and poorly understood entity.

## Conclusions

With a more proximal origin of DSA than usual and its course *through* the BP, there is a likelihood of its conflict with a neural element of the plexus, potentially leading to or worsening an existing nTOS, especially if the interscalene space is already narrowed by other congenital anomalies. There are few reports, along with our report, that support this claim. Ultrasound may reveal this dynamic conflict and guide hydrodissection of the DSA from the neural structure involved. Immediate resolution of symptoms confirms the diagnosis.

## Supplementary Information

Supplementary Material 1.

## Data Availability

Access to deidentified DICOM files of the images presented in this report and to the completed patient-reported CBSQ forms are available from the corresponding author (M.J.) upon reasonable request.

## Abbreviations

nTOS: Neurogenic thoracic outlet syndrome
MRI: Magnetic resonance imaging
DSA: Dorsal scapular artery
